# Retraction: Modeling of protein hydration dynamics is supported by THz spectroscopy of highly diluted solutions

**DOI:** 10.3389/fchem.2025.1729478

**Published:** 2025-10-31

**Authors:** Frontiers Editorial Office

The journal retracts the 09 June 2023 article cited above.

Following publication, concerns were raised regarding fundamental errors present in the methodology of this manuscript. The article does not meet the standards of editorial and scientific rigor for *Frontiers in Chemistry*; therefore, the article has been retracted.

This retraction was approved by Chief Editors of *Frontiers in Chemistry* and the Chief Executive Editor of Frontiers. The authors did not agree with this retraction. Frontiers would like to thank the concerned readers who contacted us regarding the published article.

